# When I Move, You Move: Physical Activity and Cognitive Functioning Among Older Dyads in Europe

**DOI:** 10.1177/30495334261418966

**Published:** 2026-02-04

**Authors:** Laura Himmelmann, Jeffrey E. Stokes, Tim Stuckenschneider, Tania Zieschang, Kathrin Boerner

**Affiliations:** 1 Carl von Ossietzky Universität Oldenburg, Germany; 2 University of Massachusetts Boston, MA, USA

**Keywords:** exercise, dyadic, accelerometer, cognition

## Abstract

This study investigates physical activity within dyadic relationships as a predictor of cognitive function using data from the Survey of Health, Aging, and Retirement in Europe (SHARE). A two-wave dyadic analysis was conducted for 33 opposite-sex couples (mean age 67.7 years). Physical activity was measured with a three-axial accelerometer, and cognitive function through verbal fluency at baseline and follow-up. Multilevel lagged dependent variable models were estimated using mixed effects in Stata Version 18/SE. Verbal fluency remained stable over time (β = .62, *p* < .001). Individuals’ own physical activity (steps) predicted better cognitive function at follow-up (β = .30, *p* < .001). Spouses’ physical activity also showed a positive association, though weaker (β = .25, *p* < .01). Individual activity was more strongly associated with cognitive outcomes than partner activity. However, partner effects remained significant after accounting for individuals’ own activity, indicating that dyadic influences contribute uniquely beyond personal behavior. These findings underscore the relevance of dyadic processes for cognitive health in later life. Future research should examine underlying mechanisms and evaluate couple-based interventions.

## Background

Alzheimer’s disease and related dementias (ADRD) negatively impact well-being and self-reliance among older adults (Prince et al., 2015). Due to significant cortical and subcortical dysfunction, major symptoms include a decline in memory, executive functions, and verbal fluency (Machado et al., 2018). Evidence has shown that lifestyle factors such as diet, cognitive exercise, as well as physical activity help to maintain cognitive functioning in older adults, who are at an increased risk for cognitive decline due to age (Ngandu et al., 2015). Moderate to vigorous exercise has exhibited beneficial effects on brain health in community-dwelling older adults with cognitive impairment (Baek et al., 2024; Castellote-Caballero et al., 2024; Fairchild et al., 2024). Therefore, physical activity is associated with slower cognitive decline, while it also can improve well-being and self-reliance in later life (Livingston et al., 2024).

Given that verbal fluency is among the first cognitive abilities to show decline in pathological aging, it represents a key indicator for understanding how lifestyle factors, such as physical activity, may protect cognitive health. Verbal fluency, particular semantic fluency tasks such as animal naming, are among the most widely used measures of cognitive function in aging research due to their brevity, sensitivity, and ease of administration in large-scale population studies (Marra et al., 2021; Shao et al., 2014; Vonk et al., 2020; Wright et al., 2023). These tasks require participants to generate as many as words as possible within a specific category (e.g., animals) in a limited time. Performance reflects not only lexical access and semantic memory, but also executive control processes such as mental set-shifting, updating, and inhibition (Birn et al., 2010; Henry et al., 2004). Thus, verbal fluency tasks are hybrid measures that tap into both language and executive domains, making them sensitive to range of age-related cognitive changes (Clark et al., 2009).

Alongside an individual’s own physical activity, a partner’s (i.e., a spouse’s or romantic partner’s) physical activity could play an important role in promoting an active lifestyle and fostering healthier behaviors. Both social contagion of health behaviors (Christakis & Fowler, 2013) and the social control of health behaviors within relationships (Tucker, 2002) suggest that having a physically active partner would promote greater activity engagement for an individual over time, with downstream benefits for physical and cognitive health. Studies using sensor-based activity data on older adults, however, are scarce. One previous study has shown that both physical activities spent in moderate to vigorous intensity (MVPA) and sedentary behavior were correlated in *n* = 108 couples aged 60 to 87 years. Thus, a higher MVPA synchrony was associated with a higher individual’s weekly MVPA and lower individual’s sedentary behavior (Pauly et al., 2020). Another study investigated physical activity, psychological health, and cognitive function among *n* = 4 older adults with cognitive impairment and their romantic care partners, after they participated in a dyadic exercise program. Study results indicated that a dyadic physical exercise program was helpful for romantic care partners in terms of increasing their care support, aerobic fitness and executive function, while at the same time concerns about falling decreased, and both aerobic fitness and executive function increased in older adults with cognitive impairment (Ahn et al., 2024). Given these insights, dyadic physical exercise approaches may not only be effective in improving an individual’s own but also a partner’s physical activity and thereby mitigate cognitive decline in couples. Especially in relationships where an individual relies on a spouse’s support to accomplish activities of daily living (i.e., eating, bathing, dressing, transferring, toileting, moving around), it is helpful for both the spouse and the individual to exercise together, as has been previously described in the literature (Ahn et al., 2024).

Although previous research findings, especially concerning the older population, seem promising, there is still limited research on the *longitudinal* effects of both an individual’s own and their partner’s physical activity on cognitive outcomes. That is, it is unclear whether (a) having an active partner may spur greater activity engagement and healthier behaviors in oneself, leading to better cognitive outcomes, or whether (b) in older age if particular adults’ health behaviors are stable, and one’s own – but not a partner’s – physical activity engagement would have meaningful influence on cognitive outcomes. Therefore, this study aims to investigate the extent to which both one’s own and a partner’s sensor-based physical activity levels predict maintenance of cognitive function in older individuals. Using a two-wave longitudinal design from the Survey of Health, Aging, and Retirement in Europe (SHARE), we hypothesize that both an individual’s own and a partner’s physical activity will be positively associated with cognitive trajectories in older adults. This dyadic approach could provide novel insights into the longitudinal association of couples’ physical activity and cognitive functioning in later life.

## Methods

### Sample

Participants analyzed in this study were taken from wave 8 (2019/2020) and wave 9 (2021/2022) of the Survey of Health, Aging and Retirement in Europe (SHARE), including data from Belgium, Czech Republic, Denmark, France, Germany, Italy, Poland, Spain, Slovenia, and Sweden. These waves were selected due to the availability of sensor-based physical activity data at wave 8 only, with wave nine then used to assess change in cognition over time. SHARE is a multidisciplinary pan-European study, aiming to define trajectories of health, social network, and economic conditions of aging populations. In both waves, current partners living in the same household were interviewed. The SHARE population consists of adults at least aged 50 years and older, who have their regular domicile in one of the mentioned SHARE countries. Data were collected by using computer-assisted personal interviewing, except for data collection of physical activity measures, which were collected in person. Participants gave their written informed consent prior study inclusion and data were pseudo-anonymized.

A total of 855 participants completed accelerometer data in wave 8 of SHARE. However, data collection was not originally designed to be dyadic; that is, not all participants who completed the accelerometer data collection were partnered, nor were both partners necessarily included in the accelerometer data collection module. However, among the 855 participants included in this module, a total of 80 individuals were from opposite-sex couples where both partners completed the accelerometer data collection (i.e., 40 couples with actor and partner data at wave 8). Of these, a total of 60 individuals returned at wave 9 and provided valid cognition data. Individuals were included in the sample if both they and their partner provided valid accelerometer data at baseline, and the individual him/herself provided valid cognition data at follow-up, irrespective of whether the partner provided data at follow-up as well. A total of 60 individuals, from 33 opposite-sex couples, comprise the analytic sample for this study.

### Outcome Measures

Cognitive function was measured using a verbal fluency instrument, wherein individuals were asked to name as many animals as possible in 1 min. The score is the sum of acceptable animals. Verbal fluency was measured at both wave 8 and wave 9, with the wave 8 scores serving as a lagged dependent variable (LDV) predictor of wave 9 verbal fluency, to account for between-person differences in cognition as well as for stability in verbal fluency over time.

### Predictor Measures

Sensor-based physical activity levels were assessed by the Axivity AX3 (Axivity Ltd, Newcastle upon Tyne, United Kingdom) accelerometer. Participants subsequently received the accelerometer via postal mail, together with an information letter and an illustrated instruction brochure on how to put on the device. Participants also received a prepaid return envelope to send the accelerometer and the reply card back to the respective survey agency after data collection. The device was attached to participants upper thigh by means of medical-style adhesive tape and participants were advised to wear the sensor for eight consecutive days including a 24-hr cycle. Sampling rate was set at 50 Hz with a range of ±8 g (1g = 9.81 ms^2^). Non-wear time is detected, and imputation of non-wear time is performed. Total number of steps and moderate to vigorous time spent in physical activity (MVPA) per day for the participant *and* the spouse (i.e., dyadic actor and partner effects) were used as the focal predictors for analysis.

### Statistical Analysis

We utilized dyadic multilevel LDV modeling using mixed effects models to address our research questions. Multilevel modeling accounts for the non-independence of observations due to the nesting of individuals within couples. Models were dyadic, as data from both partners were analyzed, and accelerometer data for both oneself and one’s partner were included in models as focal predictors. LDV modeling accounts for between-person stability of the outcome (verbal fluency) over time and allows for coefficients to be interpreted in terms of *change* over time. We estimated separate multilevel models to test the effects of our two distinct accelerometer predictors. All statistical models were adjusted for the following covariates: individuals’ age (in years), gender (male, female), educational attainment (ISCED classification), wealth (total household income, adjusted for median national income), and country. A total of 60 individuals nested within 33 couples comprised the final analytic sample. There was no item-missingness for measures included in the analysis, after trimming the sample to account for attrition over time.

## Results

### Participant Characteristics

 Participants were 67.7 years of age on average, ranging from 53 to 87 (see Table 1). Verbal fluency was stable across timepoints, averaging 22.4 at baseline and 22.6 two years later (*p* > .10). Participants averaged 7,813 steps overall, ranging from a minimum of 1,259 to a maximum of 17,630, and engaged in 54 min of moderate to vigorous exercise per day on average (range: 11.1–138.7).

**Table 1.** Descriptive Statistics, Measures of Interest.

Variable name	*N*	Mean	*SD*	Min	Max
Age	60	67.7	8.7	53	87
Gender, female, *n* (%)	60	32 (53.3)	-	-	-
Verbal fluency (follow-up)	60	22.6	7.2	6	39
Verbal fluency (baseline)	60	22.4	6.7	10	40
Number of steps per day	60	7,813.8	3,529.5	1,259	17,630.4
MVPA, minutes per day	60	54.3	27.9	11.2	138.7

*Note.* MVPA = activity spent in moderate to vigorous intensity; Min = minimum; Max = maximum; *SD* = standard deviation.

### Analytic Results

As anticipated, there was significant stability in verbal fluency from wave 8 to wave 9 (β = .62, *p* < .001). In both the models concerning total number of steps (β = .30, *p* < .001) and moderate to vigorous activity (*β* = .30, *p* < .01), participants’ own accelerometer activity was significantly associated with better verbal fluency at wave 9, accounting for their verbal fluency at wave 8; that is, individuals’ own physical activity was linked with slower declines to verbal fluency over time (see Tables 2 and 3). In the model concerning total steps, a spouse’s number of steps was also associated with significantly better verbal fluency at follow-up (*β* = .25, *p* < .01; see Table 2). Likewise, a spouse’s engagement in moderate or vigorous physical activity was linked at trend-level significance with better verbal fluency at follow-up (*β* = .18, *p* < .06; see Table 3).

**Table 2.** Multilevel Dyadic Model Concerning Number of Steps and Verbal Fluency.

Predictor	ß	*SE*	*z*	*p*-Value	95% CI
Verbal fluency (baseline/wave 8)	0.6706	0.0807	8.3	<.001***	[0.5124, 0.8288]
Number of steps (individual)	0.0006	0.0002	3.6	<.001***	[0.00022, 0.00094]
Number of steps (spouse)	0.0043	0.0002	2.8	.005**	[0.00013, 0.00073]

*Note. N* = 33 couples. ß = ß-coefficient; SE = standard deviation; *z* = *z*-score; 95% CI = 95% confidence interval; MVPA = activity spent in moderate to vigorous intensity. Model adjusted for individuals’ age, gender, educational attainment, wealth, and country.

Ϯ *p* < .10. *p < .05. **p < .01. ***p < .001.

**Table 3.** Multilevel Dyadic Model Concerning Moderate to Vigorous Activity and Verbal Fluency.

Predictor	ß	*SE*	*z*	*p*-Value	95% CI
Verbal fluency (baseline/wave 8)	0.6829	0.0830	8.2	<.001***	[0.5202, 0.8457]
MVPA, minutes per day (individual)	0.0784	0.0230	3.4	.001**	[0.0334, 0.1234]
MVPA, minutes per day (spouse)	0.0398	0.0210	1.9	.058Ϯ	[−0.0013, 0.0809]

*Note. N* = 33 couples. ß = ß-coefficient; SE = standard deviation; z = z-score; 95% CI = 95% confidence interval; MVPA = activity spent in moderate to vigorous intensity. Model adjusted for individuals’ age, gender, educational attainment, wealth, and country.

Ϯ *p* < .10. *p < .05. **p < .01. ***p < .001.

## Discussion

European older adults living in a dyadic relationship benefited from physical activity engagement to maintain their cognitive function over the 2-year assessment period. First, we found that individuals’ enhanced sensor-based physical activity levels were associated with slower declines in cognitive functioning, as anticipated. Second, we found that higher levels of a spouse’s engagement in physical activity were also positively correlated with individuals’ cognitive performance at follow-up, suggesting a “spillover” effect whereby having an active partner may have benefits for individuals over time as well, most likely through enhanced activity engagement oneself over time (Christakis & Fowler, 2013; Tucker, 2002). Importantly, the partner effects emerged above and beyond the individuals’ own activity levels, highlighting that a spouse’s behavior can independently contribute to cognitive maintenance. Taken together, results suggests that both *being more active* and *having a more active partner* may be protective against declines to verbal fluency in later life. Nevertheless, the associations should be interpreted as correlational and exploratory, given the limited statistical power and design constraints of the study.

These findings are particularly meaningful when considering the nature of verbal fluency as a cognitive outcome measure. Verbal fluency, especially semantic fluency tasks such as animal naming, is among the most widely used indicators of cognitive functioning in aging research. That is due to its brevity, sensitivity, and high ecological validity (Shao et al., 2014). From a psychometric perspective, verbal fluency has demonstrated robust reliability and validity across diverse populations and longitudinal. Evidence suggests that declines in semantic fluency often precede more global cognitive deterioration, making it a sensitive early marker for dementia (Sutin et al., 2019). However, several factors can influence performance, including education, cultural background, and language proficiency, which reflect cognitive reserve rather than pure ability (Kempler et al., 1998). Moreover, as the measure is frequently reduced to total word count, it may not fully capture finer distinctions between semantic memory and executive control processes. Still, because fluency performance is partially shaped by cognitively, socially, and physically stimulating behaviors, it may share overlapping mechanisms with physical activity itself (Coelho-Júnior et al., 2024).

Despite these limitations, verbal fluency remains an invaluable and sensitive cognitive indicator in large-scale population studies, where more comprehensive neuropsychological testing is not feasible. It reflects essential domains, executive efficiency, lexical retrieval, and processing speed that have consistently been shown to benefit from physical activity interventions (Colcombe & Kramer, 2003; Stillman et al., 2020). Several studies have reported that higher levels of physical activity, particularly moderate-to-vigorous exercise, are associated with better fluency performance in older adults (Etnier et al., 1997; Netz, 2019). For instance, moderate-intensity exercise and dual-task training combining movement and word generation have both been linked to improvements in executive control and fluency (Chen et al., 2023; Jardim et al., 2021). Thus, verbal fluency appears not only theoretically connected to neural systems sensitive to exercise, such as prefrontal and temporal regions, but also empirically responsive to physical activity interventions.

In this context, our findings suggest that the observed associations between dyadic physical activity and verbal fluency trajectories are grounded in well-established psychometric and neurocognitive principles. At the same time, the small sample size and the exploratory nature of the analyses require that any directional interpretation be treated cautiously. Alternative explanations, such as shared lifestyle environments, assortative matching (i.e., partners naturally selecting each other based on similar health behaviors), or unmeasured dyadic confounders may also contribute to the observed patterns. By leveraging verbal fluency as a theoretically justified and sensitive proxy of cognitive functioning, our study provides a valuable contribution by demonstrating that both an individuals’ and partners’ physical activity predict cognitive outcomes longitudinally, emphasizing the role of interpersonal influences in older adults’ cognitive aging.

Our findings are in line with results from Pauly et al. (2020), who reported when spouses engaged in more MVPA than usual, the individual was more likely to increase one’s own physical activity behavior. The authors suggested that perhaps individuals and their spouse acted as role models for one another concerning health-related behaviors, thereby increasing their physical activity over time. This aligns with both social contagion theory (Christakis & Fowler, 2013) and social control theory (Tucker, 2002). By showing that partner effects were observed for MVPA, our results suggest that certain types of physical activity may be more socially transmissible or salient within dyadic contexts, providing insight into which behaviors could be targeted in interventions.

In contrast to the insights provided by Pauly and colleagues, our study extends previous findings by not only analyzing physical activity behavior, but by incorporating the association of physical activity behavior and cognition over a 2-year span among older couples. Further, other recent studies in this area have only investigated whether physical exercise programs may stimulate cognitive function, and have focused on populations of adults with cognitive impairment or dementia such as Alzheimer’s disease and their caregivers (Ahn et al., 2024; Mehling et al., 2020). Whether and to what extent dyadic partners mutually influence each other with their engagement in physical activity remains unclear. Moreover, if physically active individuals always cohabit with likewise active counterparts and therefore do not need to influence each other at all on a behavioral level, and whether this has a positive effect on cognitive performance in general remains also unclear. Another study investigated the efficacy of a community-based exercise program in *n* = 30 dyads of older adults with mild to moderate dementia, who participated together with their care partners, on several cognitive and health-related outcomes (Mehling et al., 2020). Contrary to the findings reported by Ahn et al., the dyadic exercise program did not reveal a meaningful change, neither on physical performance, nor on cognitive outcome measures (Mehling et al., 2020). Since the optimal form and intensity of exercise have not yet been identified and may vary based on individual characteristics, the exercise stimulus in the intervention by Mehling et al. may have been insufficient. Therefore, future studies should aim to control and report exercise intensity to ensure an adequate exercise stimulus, ideally with larger sample sizes. In contrast, the present study leverages a real-world community-based sample drawn from the SHARE study, a large, multidisciplinary, pan-European dataset that, while not originally designed for dyadic accelerometer-based analyses, provides rare sensor-based physical activity data from both partners at two time points. This design inevitably led to substantial data loss due to the requirement that both partners wear accelerometers at both waves, but it also underscores the uniqueness and value of the available dyadic sensor-based physical activity data. Despite the small sample size, however, our findings build upon this extant research by indicating that *both partners’* physical activity engagement was influential for cognitive functioning (a) in the absence of a structured intervention, and (b) among community-dwelling older adults not yet afflicted with substantial cognitive impairment. Overall, these results highlight the importance of considering dyadic processes when studying cognitive aging, as the longitudinal design controlling for baseline cognitive function demonstrates that partner behaviors can exert an independent, lasting influence on cognitive trajectories.

Given the limited number of trials and the inconsistent findings in studies examining the impact of exercise programs on cognition and functional performance in dyads of older adults, it is crucial to first gain a deeper understanding of the relationship between physical activity and cognitive performance in these dyadic relationships. Our findings therefore should be interpreted as preliminary, exploratory evidence rather than confirmatory proof of dyadic causal pathways. That is, interventions targeting couples may be more beneficial than those targeting individuals alone. Future studies should investigate the mechanisms underlying the observed long-term protective effects of physical activity within dyadic relationships on cognitive performance. Moreover, expanding this research to include diverse demographic and cultural as well as different age groups could provide a more comprehensive understanding of how dyadic dynamics influence cognitive health in aging populations. Future work should also explore why partner effects are stronger for some types of activity than others, and whether these effects translate into protection across multiple cognitive domains. Such insights could help develop programs that encourage couples to act as role models for each other’s health behaviors, fostering physical activity and supporting cognitive health in later life.

### Limitations

Several limitations should be considered when interpreting the findings of this study. First, although the use of sensor-based physical activity measures represents a methodological strength compared to self-reported assessments, the analytic sample was reduced due to substantial attrition and missing accelerometer data, which may have limited statistical power and reduced the generalizability of results. This issue is partly attributable to the design of the SHARE dataset: because dyadic analyses required that both partners wore accelerometers at both waves, thus many cases had to be excluded. While this contributed to the small sample size, it also highlights the rarity and value of dyadic sensor data in large-scale population studies. Second, the relatively small sample size restricts the ability to detect smaller effects and to examine subgroup differences, such as gender or age-related variations in dyadic dynamics. Accordingly, all findings should be interpreted as exploratory. Third, cognitive functioning was operationalized solely through verbal fluency, which, while psychometrically robust and sensitive to age-related change, does not fully capture the multidimensional nature of cognitive functioning. More comprehensive cognitive batteries assessing domains such as working memory, processing speed, and reasoning would provide a richer understanding of how physical activity relates to cognition in dyadic contexts. Fourth, although item-missingness was largely non-existent in this analytic sample, future research would benefit from explicitly incorporating imputation strategies or conducting sensitivity analyses including individuals and couples lost to non-compliance with actigraphy data collection, to assess the robustness of results across missing-data assumptions. Finally, although the longitudinal design allowed controlling for baseline cognitive levels, the observed effects are limited to verbal fluency and should be interpreted cautiously when generalizing to other cognitive domains. And finally, while we controlled for age within our statistical model, the large age range in our sample suggests that forming distinct age groups could be beneficial for analyzing dyadic relationships and sensor-based physical activity levels more precisely. However, this approach would require a sufficiently larger sample size than the one analyzed in our study, to ensure reliable subgroup analyses. Therefore, the present results should be interpreted with caution, and replication in larger samples using more diverse cognitive measures is warranted to confirm the robustness and generalizability of these findings.
